# Bioinspired Electropun Fibrous Materials Based on Poly-3-Hydroxybutyrate and Hemin: Preparation, Physicochemical Properties, and Weathering

**DOI:** 10.3390/polym14224878

**Published:** 2022-11-12

**Authors:** Polina M. Tyubaeva, Ivetta A. Varyan, Anna K. Zykova, Alena Yu. Yarysheva, Pavel V. Ivchenko, Anatoly A. Olkhov, Olga V. Arzhakova

**Affiliations:** 1 Academic Department of Technology and Chemistry of Innovative Materials, Plekhanov University of Economics, Stremyanny Per. 36, Moscow 117997, Russia; 2 Department of Biological and Chemical Physics of Polymers, Emanuel Institute of Biochemical Physics, Russian Academy of Sciences, ul. Kosygina 4, Moscow 119334, Russia; 3 Faculty of Chemistry, Lomonosov Moscow State University, Leninskie Gory 1/3, Moscow 119991, Russia

**Keywords:** biodegradable polymers, poly(3-hydroxybutyrate), hemin, electrospinning, outdoor weathering, new technologies

## Abstract

The development of innovative fibrous materials with valuable multifunctional properties based on biodegradable polymers and modifying additives presents a challenging direction for modern materials science and environmental safety. In this work, high-performance composite fibrous materials based on semicrystalline biodegradable poly-3-hydroxybutyrate (PHB) and natural iron-containing porphyrin, hemin (*Hmi*) were prepared by electrospinning. The addition of *Hmi* to the feed PHB mixture (at concentrations above 3 wt.%) is shown to facilitate the electrospinning process and improve the quality of the electrospun PHB/*Hmi* materials: the fibers become uniform, their average diameter decreases down to 1.77 µm, and porosity increases to 94%. Structural morphology, phase composition, and physicochemical properties of the *Hmi*/PHB fibrous materials were studied by diverse physicochemical methods, including electronic paramagnetic resonance, optical microscopy, scanning electron microscopy, and energy-dispersive X-ray spectroscopy, elemental analysis, differential scanning calorimetry, Fourier-transformed infrared spectroscopy, mechanical analysis, etc. The proposed nonwoven *Hmi*/PHB composites with high porosity, good mechanical properties, and retarded biodegradation due to high antibacterial potential can be used as high-performance and robust materials for biomedical applications, including breathable materials for wound disinfection and accelerated healing, scaffolds for regenerative medicine and tissue engineering.

## 1. Introduction

At the present time, the mainstream direction of modern materials science and technology is oriented toward the development of innovative and ecologically friendly materials based on natural components and biodegradable polymers with task-oriented multifunctional properties presents. In this connection, the family of biodegradable polyhydroxyalkanoates, including poly(hydroxybutyrate) (PHB) is of special interest due to their potential as biomedical materials for therapeutic applications. PHB seems to be a promising candidate for the development of composite materials for biomedical applications as this biopolymer is characterized by high melting temperature, high degree of crystallinity, and low permeability to oxygen, water, and carbon dioxide [1]. This biodegradable polymer can be synthesized from renewable natural sources and is known to be biocompatible with the human body [2].

The introduction of various additives to PHB allows the preparation of the composite PHB-based materials with desired functional properties (nanoparticles [3], carbon nanotubes [4], catalysts, and enzymes [5]). Nowadays, special interest is focused on the molecular complexes of porphyrins and related materials for their applied use as superconductors [6,7,8], in analytical chemistry [9,10], photonics [11,12], gene therapy [13,14], biomedicine [15,16,17]. Progress in the synthesis and chemistry of tetrapyrroles has triggered the ever-growing attention to tetrapyrrole complexes as an effective modifying additive for polymeric materials, including numerous analogs of natural systems such as porphyrins, texaphyrins, chlorins, corroles, and others, applied for biomedical purposes [18,19,20,21,22].

Hemin is the iron-containing porphyrin, which is endogenously produced in the human body, for example, during the turnover of old red blood cells. The unique properties and benefits of hemin have been widely discussed in the literature [23,24,25,26,27,28,29,30,31,32]. Evidently, hemin is biocompatible with living organisms [23] and can be used for the stabilization of protein molecules on polymer carriers, and hemin-containing materials can be successfully used for diverse biomedical applications [24], including, for example, the development of safe containers for drug delivery systems [25]. Moreover, peptides can be attached to the surface of hemin-containing materials [26,27]. Hemin shows a well-pronounced catalytic activity [28] and can be effectively used in anticoagulants [29]. Hemin is characterized by high thermal stability, thus lifting the challenging problems related to the processing of polymers and the preparation of sustainable polymer nanocomposite materials [30]. In addition, hemin also shows high antimicrobial activity against Gram-positive *S. aureus* [31], which is a significant advantage in the creation of biomedical materials. Finally, the benefits of hemin are concerned with its natural origin biocompatibility with living organisms, and simple and facile procedure of synthesis [32]. Hence, hemin was selected as an efficient additive for the modification of the PHB materials and preparation of new *Hmi*/PHB materials with task-oriented functional properties.

Electrospinning (ES) is credited as a reliable and efficient method for the preparation of high-performance nonwoven materials based on a wide range of polymers [33]. This method provides the formation of an interconnected network of ultrathin fibers by drawing a jet of the polymer solution under the action of physical forces. Electrospinning offers powerful tools for the preparation of diverse composite materials when a functional additive is dissolved in the polymer feed solution [34,35,36,37]: poly(3-hydroxybutyrate-co-3-hydroxyvalerate)/polylactide [38], poly(butylene adipate succinate)/polylactide [39], poly(butylene adipate terephthalate)/polylactide [40], poly(ε-caprolactone)/poly(3-hydroxybutyrate) [41]. This ES approach allows one to control the content of additives in the composite, phase composition, and distribution of the additive within the fibrous materials.

This work addresses the development of a facile and sustainable method for the preparation of high-performance innovative biocompatible *Hmi*/PHB materials by electrospinning, characterization of their morphology, phase composition, and mechanical properties by diverse physicochemical methods (electronic paramagnetic resonance (EPR), optical microscopy, scanning electron microscopy (SEM) and energy-dispersive X-ray spectroscopy (EDX) elemental analysis, differential scanning calorimetry (DSC), Fourier-transformed infrared (FTIR) spectroscopy, mechanical analysis, etc.), investigation of their stability upon outdoor weathering tests, and description of the potential applied benefits of the resultant *Hmi*/PHB materials for diverse applications.

## 2. Materials and Methods

### 2.1. Materials

**Figure 1:**
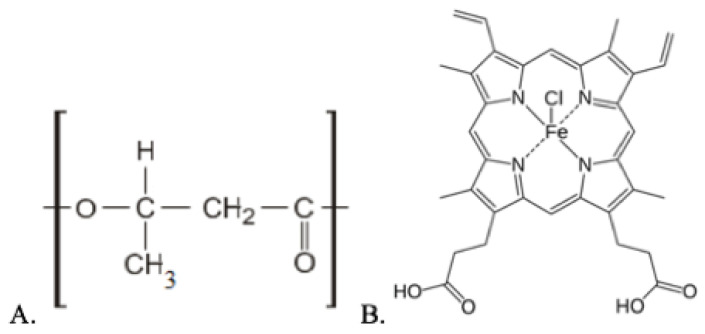
Structural formulae of PHB (**A**) and hemin (**B**).

In this work, semicrystalline biodegradable polymer, poly(3-hydroxybutyrate) (PHB) (trade mark 16F series, BIOMER, Frankfurt, Germany) was used; molecular weight—206 kDa, density—1.5 g/cm^3^ and degree of crystallinity—59% (Figure 1A). Hemin (a tetrapyrrole complex from the class of natural porphyrins) was used as a modifying additive (Figure 1B). Hemin with the coordination complex of iron (oxidation state III) was obtained from the bovine (Aldrich Sigma, Saint Louis, MO, USA).

### 2.2. Preparation of Electrospun Materials

In this work, polymer nanofibrous materials were prepared by electrospinning (ES) technique using a single-capillary laboratory unit with a capillary diameter of 0.1 mm (EFV-1, Moscow, Russia) (Supplementary Files, Figure S1). As a result of electrospinning, polymer fibers are cured due to the complete evaporation of the solvent. For the preparation of feed solutions, finely dispersed PHB powder was dissolved in chloroform at a temperature of 60 °C. Hemin was dissolved in N,N-dimethylformamide at 25 °C and homogenized with the PHB solution. The content of PHB in the solution was 7 wt.%, and the content of hemin was 1, 3, and 5 wt.% of the PHB. The conditions of the ES process were the following: voltage 17–20 kV, the distance between the electrodes—190–200 mm, the gas pressure on the solution—10–15 kg(f) cm^−2^. The electrical conductivity of the feed solution was 10–14 μS cm^−1^ and the viscosity of the hemin/PHB solution was 1.4–1.9 Pa s (viscosity of the 7 wt.% PHB solution in chloroform was 1 Pa s) and the flow rate of the solution was 0.15–0.20 cm^3^ min^−1^ (flow rate of the 7 wt.% PHB solution in chloroform was 0.45 cm^3^ min^−1^).

### 2.3. Methods

#### 2.3.1. Optical Microscopy

Primary data on topography and the general appearance of the samples were collected using an Olympus BX43 optical microscope (Tokyo, Japan) in transmission mode.

#### 2.3.2. Scanning Electron Microscopy (SEM)

The morphology of the samples was examined by SEM observations using a Tescan VEGA3 microscope (Wurttemberg, Czech Republic). Prior to SEM observations, the surface of the test samples was decorated with a thin platinum layer.

#### 2.3.3. Estimation of Structural Parameters

The morphology of the fibrous materials was studied using the Olympus Stream Basic software (Tokyo, Japan). The average diameter of individual fibers was estimated for each fiber on the z-stack at five different points. The density δ of the samples reads as:

(1) $$\delta =\frac{m}{l\times B\times b}$$

where *m* is the weight of the sample; *l* is the length; *B* is the width; *b* is the thickness.

The porosity of the fibrous electrospun materials was estimated as the ratio between the volume of pores and the overall volume of the sample:

(2) $$W=\left(1-\frac{V_{f}}{V_{s}}\;\right)\times 100,\;\%$$

where *W* is the porosity of the nonwoven sample, *V_f_* and *V_s_* stand for the volume of polymer as fibers in the nonwoven sample and the nonwoven sample, respectively.

#### 2.3.4. Energy-Dispersive X-ray Spectroscopy (EDX)

The elemental composition of the samples (oxygen, chlorine, and iron) was studied by the method of energy-dispersive X-ray spectroscopy (EDX) using a Tescan VEGA3 instrument (Wurttemberg, Czech Republic).

#### 2.3.5. FTIR Spectroscopy

The chemical composition of initial, modified, and weathered materials were studied using an FTIR Lumos BRUKER (Berlin, Germany) spectrometer at a temperature of (22 ± 2) °C in the range of wavenumbers of 4000 ≤ ν ≤ 600 cm^−6^ in the mode of frustrated total internal reflection (FTIR) on the diamond crystal [42]. The intensity of several absorption peaks related to polymer degradation (functional groups) was estimated [43,44].

#### 2.3.6. Differential Scanning Calorimetry (DSC)

The thermal properties of the test samples were studied using a Netzsch 214 Polyma thermal analyzer (Selb, Germany); the heating rate was 10 °C/min. The samples were heated from 20 °C to 220 °C and then cooled down to 20 °C. The heat of fusion was calculated using the NETZSCH Proteus software (Selb, Germany). The weight of the samples was ~7 mg. The data were analyzed according to the standard procedure [45]. The degree of crystallinity of the samples *χ* reads as:

(3) $$\chi =\Delta H_{m}/\Delta H_{m,100\%}\times 100\%$$

where Δ*H_m_* is the heat of fusion; Δ*H_m,_*_100%_ is the heat of fusion of the ideal crystal of PHB (146 J/g [46]).

#### 2.3.7. Electronic Paramagnetic Resonance (EPR)

The EPR spectra were collected using an EPR-V automated spectrometer (Moscow, Russia). Microwave power in the resonator cavity was below 7 mW. Stable nitroxyl radical (2,2,6,6-tetramethyl piperidine-1-oxyl (TEMPO) radical moiety) was used as a probe. The TEMPO radical was introduced into the polymer samples from the gas phase at 60 °C. The content of the TEMPO radicals in the polymer samples was estimated using Bruker WinEPR and SimFonia software (Berlin, Germany) (CCl_4_ with the radical concentration below 10^−3^ mol L^−1^ as a reference). Correlation times of the probe rotation were estimated from the corresponding EPR spectra.

The EPR spectra of the TEMPO spin probe in the slow-motion region (τ > 10^−10^ s) were analyzed in the terms of the model of isotropic Brownian rotation using the NLSL software [47]. Correlation times of the probe rotation τ in the fast-motion region (5 × 10^−11^ < τ < 10^−9^ s) were estimated from the corresponding EPR spectra as:

(4) $$\tau =6.65\times 10^{-10}\left(\sqrt{I_{+}/I_{-}}-1\right)\times \Delta H_{+}$$

where Δ*H_+_* is the half-width of the spectrum component in the weak field, and *I_+_/I_−_* is the ratio of the intensities of the components in weak and strong fields, respectively [48].

#### 2.3.8. Mechanical Tests

Mechanical properties (tensile strength, elongation at break) of the test samples were estimated using a Devotrans DVT GP UG 5 tensile compression testing machine (Istanbul, Turkey) according to ASTM D5035-11.

#### 2.3.9. Weathering Experiments upon Outdoor Exposure

The weathering experiments were performed according to the ASTM G7/G7M-21 (“Standard Practice for Natural Weathering of Materials”). The samples were subjected to outdoor exposure according to standard protocol D1435-13 (“Standard Practice for Outdoor Weathering of Plastics”). According to the protocol, the wooden weathering racks were tilted at an angle of 90° with respect to the horizontal line.

The dimensions of the samples were 40 mm × 40 mm × 0.3 mm (length, width, and thickness, respectively). Similar samples were placed as blank samples in a dark and dry place. The specimens were fastened with white cotton threads; one side of the specimens was fastened with adhesive tape. The duration of each weathering experiment was 3 months (June–August; temperature average 21 °C; humidity average 75.7%; geographical location: 55° 45′ north latitude; 37° 37′ east longitude).

#### 2.3.10. Statistical Analysis

All measurements were performed, at least, three times for each sample. Mechanical tests, weathering experiments, and measurements of structural characteristics were performed for 8–10 samples. A mean value and standard deviation were estimated using Data Analysis by the Origin Pro program (OriginLab Corporation, Northampton, MA, USA).

## 3. Results

Electrospinning is a well-known and reliable procedure for the development of a fibrous structure [49,50]. This method makes it possible to control the supramolecular structure and morphology of individual fibers as well as the morphology of the system as a whole [51]. The introduction of modifying additives even at low concentrations (below 5 wt.%) provides a marked effect on the formation of the structure of the resultant materials at all structural levels.

### 3.1. Morphology Structure and Properties of Hemin/PHB Fibrous Materials

**Figure 2:**
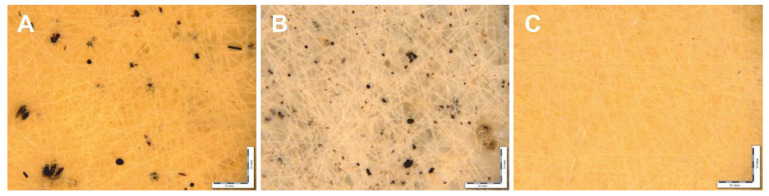
The micrographs of *Hmi*/PHB fibrous nanocomposites with different content of hemin: 1 wt.% (**A**), 3 wt.% (**B**), and 5 wt.% (**C**).

Nonwoven materials prepared by electrospinning are characterized by well-developed morphology which is described by several key parameters, including density, the average diameter of individual fibers, porosity, and pore dimensions. Figure 2 shows the optical micrographs illustrating the visual appearance of the *Hmi*/PHB samples with different content of *Hmi*.

As follows from Figure 2, the *Hmi*/PHB fibrous samples contain big-sized dark inclusions which are accommodated on the surface of individual fibers of the nonwoven sample (Figure 2A,B). The dimensions of the inclusions are equal to 4–32 µm and 0.7–17 µm for *Hmi*/PHB containing 1 and 3 wt.% of *Hmi*, respectively. Noteworthy is that the *Hmi*/PHB samples containing 5 wt.% *Hmi* are seen to be uniform and free of big-sized inclusions (Figure 2C). This fact indirectly suggests that electrospinning from the feed solutions containing 3–5 wt.% of *Hmi* is enhanced and provides the preparation of more uniform nonwoven *Hmi*/PHB materials. Analysis of the micrographs in Figure 2 allows estimation of the average diameter of individual fibers of the *Hmi*/PHB nonwoven materials. With increasing the content of *Hmi* to 5 wt.%, the average diameter of the fibers decreases from 3.5 µm for the neat PHB down to 1.77 µm for the *Hmi*/PHB containing 5 wt.% of *Hmi*. Noteworthy is that, as the content of *Hmi* in the *Hmi*/PHB composites increases, porosity of nonwoven materials increases from 80% (for neat PHB) to 94%. Hence, this evidence allows one to conclude that electrospinning of the feed solutions with the maximum content of *Hmi* leads to the preparation of nonwoven *Hmi*/PHB materials with uniform structure and high porosity. Structural characteristics of the nonwoven *Hmi*/PHB materials are listed in Table 1. The histogram illustrating the size distribution of PHB and *Hmi*/PHB nonwoven composites is given in the Supplementary Files, Figure S2.

Hence, the presence of 5 wt.% *Hmi* in the *Hmi*/PHB feed mixture is associated with a lower density of the feed solution and facilitates the ES process due to better-organized movement through the jet. As a result, pore size decreases, and porosity increases. This is due to the fact that the diameter of the PHB fibers is higher than that of the Hemin-modified fibers. Thicker PHB fibers are also characterized by the presence of large defects and their diameter exceeds the average diameter of the fibers by 3–10 times. As a result, upon ES, thicker fibers are located at a higher distance from each other, forming large pores, and this effect is the consequence of low electrical conductivity of the spinning solution. However, as Hemin is added into the feed solution, the number of big-sized defects on the surface of fibers decreases, the diameter of fibers is reduced, thinner fibers are packed in a more uniform mode, and the distance between the neighboring fibers is smaller, thus leading to smaller pores. In other words, when the density of the material is reduced (Table 1), the resultant material is characterized by a higher content of air layer between well-cured fibers without bonds, smudges, and thicker regions.

**Figure 3:**
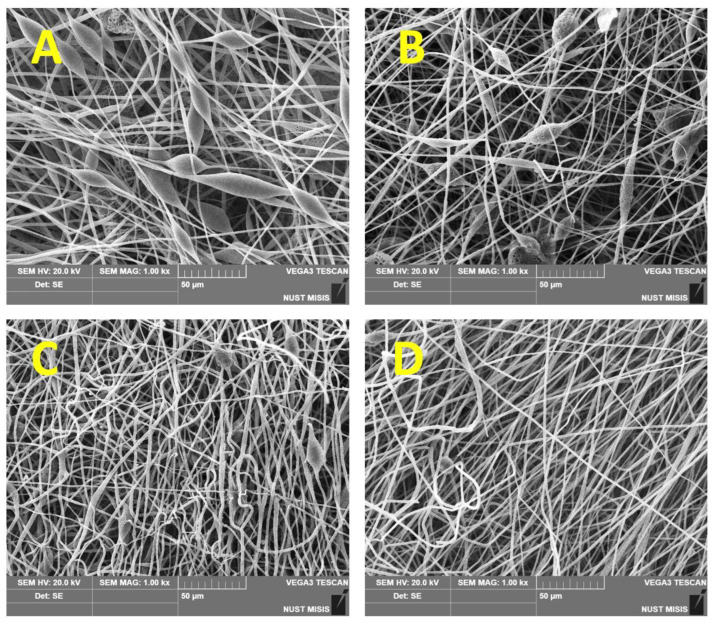
SEM micrographs of PHB and *Hmi*/PHB nonwoven materials with different content of *Hmi*: (**A**) neat PHB, (**B**) *Hmi*/PHB with 1 wt.% of *Hmi*, (**C**) *Hmi*/PHB with 3 wt.%, (**D**) *Hmi*/PHB with 5 wt.% of *Hmi*.

The fine structure of the fibrous *Hmi*/PHB materials was studied in more detail by SEM observations. Figure 3 shows the corresponding SEM images. As follows from Figure 3, the fibrous materials based on neat PHB and *Hmi*/PHB with a low content of *Hmi* (below 3 wt.%) are seen to be non-uniform; the fibers contain joints, crimps, and big-sized elements as spindles. The average size of structural imperfections ranges between 20 and 30 μm along the longitudinal direction and between 15–25 μm in the perpendicular direction. With increasing the content of *Hmi* in the *Hmi*/PHB system, the diameter of fibers markedly decreases, and the fibers within nonwoven materials are seen to be more uniform. Noteworthy is that, for the *Hmi*/PHB systems containing 5 wt.% of *Hmi*, the individual fibers are nearly free of structural defects and imperfections. Both roughness on the fiber surface almost completely, and spindle-shaped thickenings disappeared at 5 wt.% content of Hemin.

**Figure 4:**
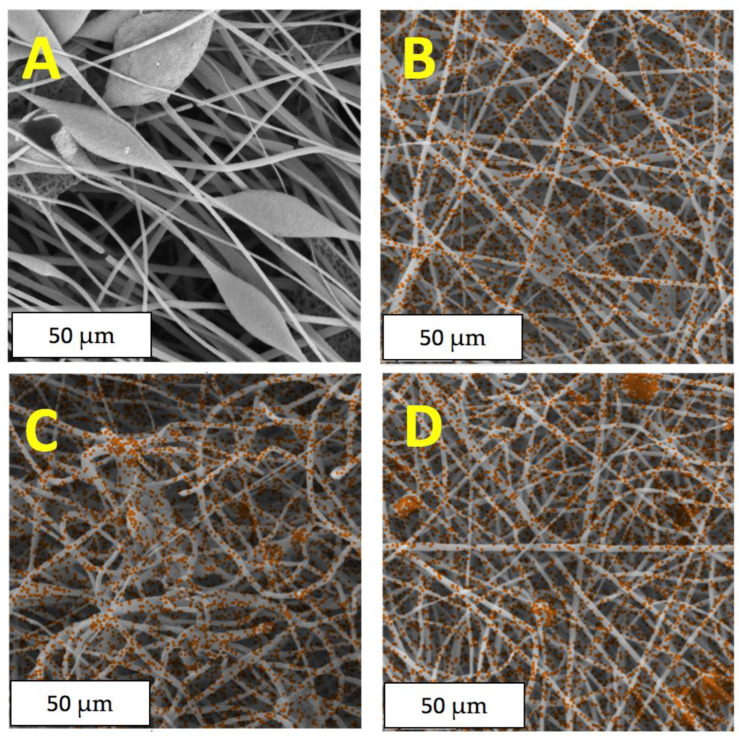
EDX elemental analysis of iron atoms in the *Hmi*/PHB samples with different content of hemin: (**A**) 0 wt.%, (**B**) 1 wt.%, (**C**) 3 wt.%, (**D**) 5 wt.%.

To gain a deeper insight into the fine structure of the *Hmi*/PHB materials, elemental analysis, and distribution of *Hmi* additive within the fibers, the *Hmi*/PHB materials were studied by the EDX method (Figure 4).

According to the EDX analysis, hemin is incorporated into fibers and uniformly distributed within the polymer structure on the surface and in the bulk. Iron and chlorine atoms serve as probes for the characterization of the distribution of *Hmi* within the fibers as the above atoms are the coordination centers of the tetrapyrrole ring. Noteworthy is that the distribution of iron and chlorine atoms appears to be identical (hence, the EDX micrographs illustrating the presence of iron atoms is presented). In Figure 4, iron atoms are seen as orange dots. In the case of the neat PHB, neither iron nor chlorine atoms are seen.

As was mentioned above, PHB is a semicrystalline polymer, which is composed of amorphous and crystalline phases. The crystalline phase is presented by lamellae with folded chains [52]. Under certain conditions formation of spherulites is also allowed [53]. The amorphous phase exists in the rubbery state (glass transition temperature of PHB is 7 °C) The influence of technological methods was very great in the formation of the crystal structure of PHB, since it introduced boundary conditions in the process of formation of the supramolecular structure. Thus, the ES method promoted the formation of pass-through oriented macromolecules [54].

**Figure 5:**
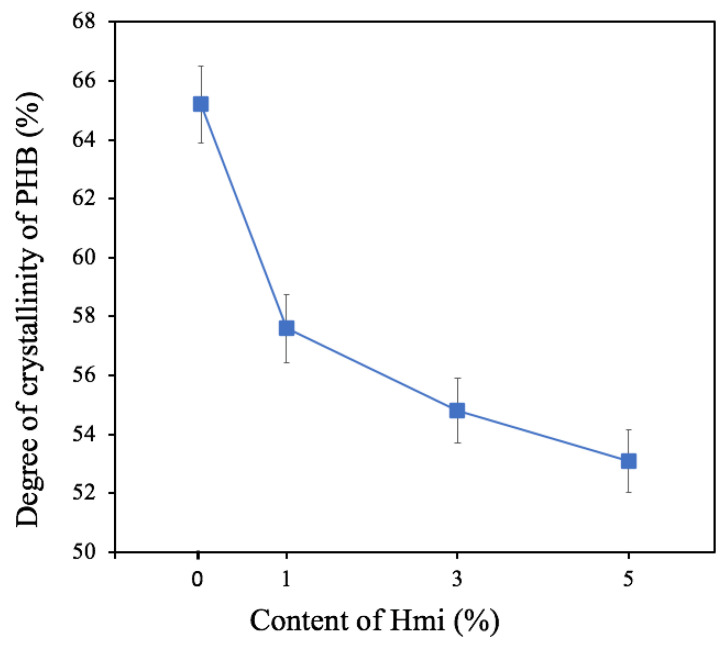
Degree of crystallinity versus the content of *Hmi* in the *Hmi*/PHB system.

According to the DSC tests, the degree of crystallinity of PHB is ~60–65%. Hence, the content of the amorphous phase in the rubbery state is ~35–40% [55]. As follows from Figure 5, as the content of *Hmi* in the *Hmi*/PHB system increases to 5 wt.%, the degree of crystallinity decreases by 12%. Hence, the presence of *Hmi* has a certain effect on the crystallization of PHB from the solution during the ES process.

**Figure 6:**
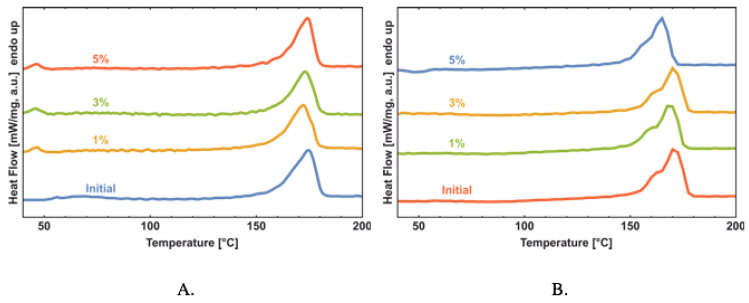
The DSC scans of Hmi/PHB with different content of Hmi: first heating run (**A**), second heating run (**B**).

Of special interest are the profiles of the melting DSC runs curve for the *Hmi*/PHB samples with the different content of *Hmi*. As the content of *Hmi* in the *Hmi*/PHB materials increases, the melting peak upon the first heating run (Figure 6A), becomes more symmetric, the melting temperature remains virtually unchanged, and the degree of crystallinity decreases. Hence, this evidence makes it possible to conclude that, in the presence of *Hmi*, crystallization of PHB proceeds at a slower rate and in a more uniform manner thus leading to the formation of a more regular and well-organized structure. Noteworthy is that, for the samples with a higher *Hmi* content, the low-temperature shoulder in the temperature interval of 150–160 °C becomes less pronounced, thus indicating the lower content of poorly organized crystallites and defects. On the second heating run, the DSC scans show double melting peaks, and this behavior is typical of PHB and corresponds to the melting-recrystallization-remelting process. The melting temperature of initial crystals is observed at 160 °C, whereas the melting temperature after recrystallization is about 174 °C.

**Figure 7:**
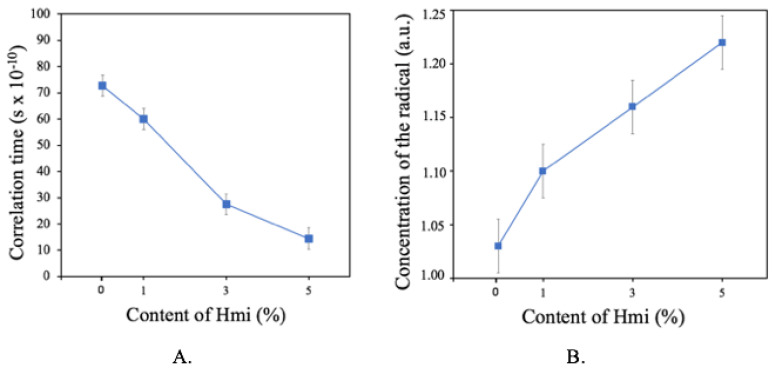
Correlation times (**A**) and concentration of TEMPO (**B**) versus the content of *Hmi* in the *Hmi*/PHB material.

The Hmi/PHB materials were studied by the EPR method to gain a deeper insight into the inner structure of the amorphous regions of PHB. Noteworthy is that the spin probe radical TEMPO is localized only within the amorphous phase of semicrystalline PHB whereas the crystalline regions contain no TEMPO. Hence, the analysis of the corresponding EPR spectra provides information concerning the mobility of spin radicals within the amorphous regions. Figure 7 shows the correlation times and concentration of TEMPO radicals plotted against the content of *Hmi* in the samples. As follows from Figure 7A, as the content of *Hmi* in the *Hmi*/PHB samples increases, the correlation time tends to decrease, and this decrease suggests the mobility of the TEMPO spin probe is reduced. The content of the spin probe (Figure 7B) appears to be independent of the *Hmi* content in the *Hmi*/PHB samples [54]. In other words, even when the concentration of the probe radical is the same, the mobility of the spin probes decreases.

Marked changes in supramolecular structure and morphology of the *Hmi*/PHB lead to changes in the physical-mechanical properties of the resultant materials (Table 2). For the *Hmi*/PHB containing 5 wt.% of *Hmi*, tensile strength increases up to 5.5 MPa, and this value is three times higher than that of the PHB nonwoven materials. Elongation at break is equal to 6% (1.7 times higher than that of the initial PHB).

### 3.2. Outdoor Exposure of Hemin/PHB Fibrous Materials

**Figure 8:**
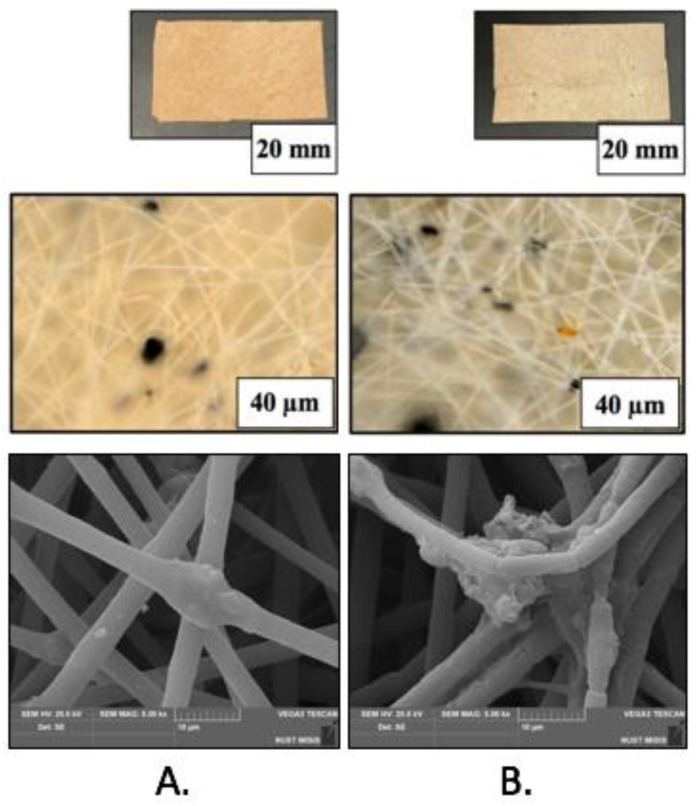
Snapshots and SEM images of the *Hmi*/PHB samples before (**A**) and after (**B**) outdoor exposure.

The stability of the biodegradable *Hmi*/PHB materials containing 5 wt.% of *Hmi* was studied under outdoor exposure. The microscopic images of the test samples before and after weathering experiments are shown in Figure 8. In contrast to the PHB nonwovens containing porphyrins with metal ions when degradation within 60–90 days is complete, the *Hmi*/PHB materials remain nearly intact: the material retained its integrity and shape.

**Figure 9:**
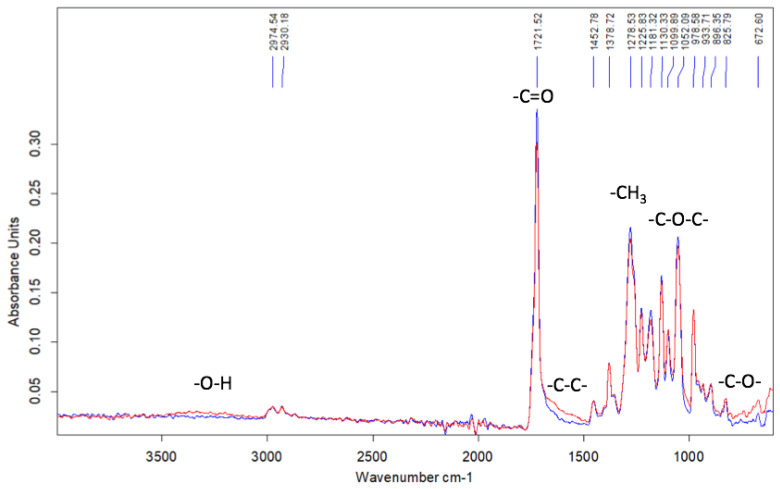
The FTIR spectra of pristine (blue line) and weathered (red line) *Hmi*/PHB samples.

The corresponding FTIR spectra of the initial and weathered samples are seen to be nearly the same and reveal no presence of the accumulated products of the degradation process (Figure 9).

The FTIR spectrum shows a slight decrease in the intensity of the peaks at 1721 cm^−1^ (C=O group), 1278 cm^−1^ (CH_3_ group), and 1052 cm^−1^, (C-O-C group). In the region of 900–700 and 1700–1500 cm^−1^, noticeable changes are observed. This fact suggests the breakage of C-C bonds and the accumulation of OH groups.

**Figure 10:**
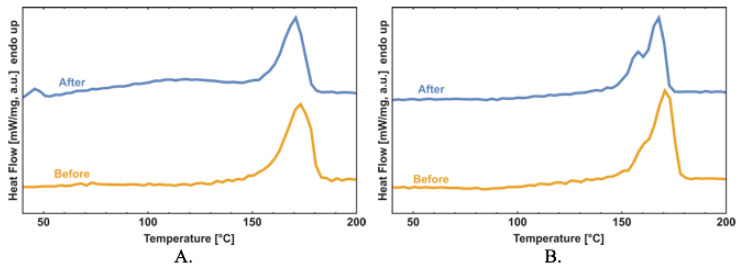
DSC scans of hemin/PHB (5 wt.%) nanocomposites before (yellow line) and after weathering (blue line): (**A**) first heating run and (**B**) second heating run.

Even though the weathered *Hmi*/PHB samples experience minor biodegradation, their supramolecular structure after weathering is changed. The results of the DSC analysis are presented in Figure 10. The shape of the melting peak is markedly changed, thus indicating the occurrence of certain processes during the exposure period.

The heat of fusion (Δ*H*) and melting temperature (T_m_) of intact and weathered PHB and *Hmi*/PHB (5 wt.%) nanocomposites are listed in Table 3.

**Figure 11:**
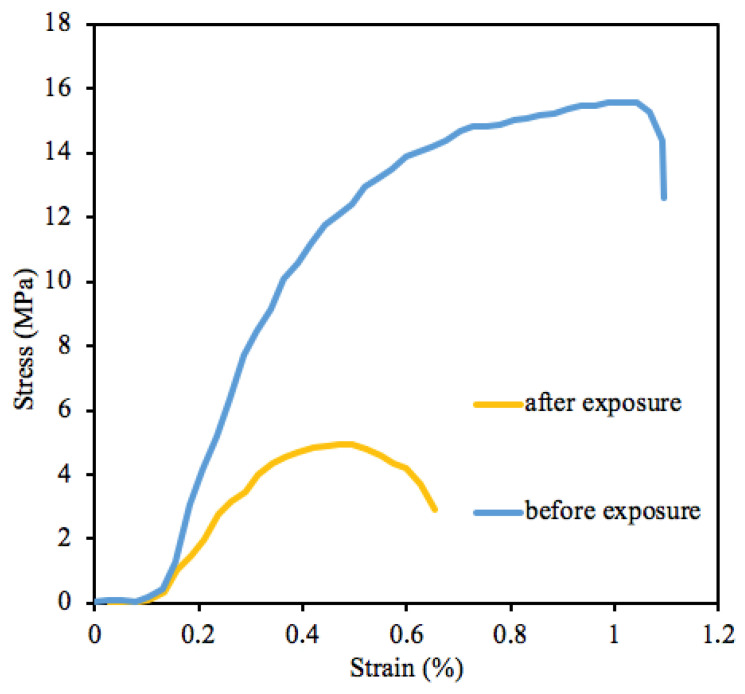
Strain-stress curves of the initial (blue line) and weathered (yellow line) samples.

The corresponding stress-strain curves of the *Hmi*/PHB containing 5 wt.% of *Hmi* before and after weathering are shown in Figure 11.

As follows from Figure 11, upon weathering, the mechanical characteristics (tensile stress and elongation at break) are markedly deteriorated. Information on tensile strength and elongation at break is presented in Table 4.

Upon weathering, the tensile strength of the *Hmi*/PHB materials decreases by a factor of 2, and elongation at break decreases from 6.1 down to 4.2%. However, even after outdoor exposure, the *Hmi*/PHB materials (in contrast to the weathering of the neat PHB) preserve their stability and high mechanical properties. Reduced mechanical properties suggest the occurrence of certain irreversible destruction processes promoted by the photosensitizing activity of the tetrapyrrole complex of Hemin. However, the samples preserve good mechanical properties, and both strength and elongation are higher than those of the initial PHB. Slightly increased strength characteristics of PHB are known to be provided by oxidation and UV irradiation.

## 4. Discussion

Nonwoven materials are characterized by three hierarchical levels of structural organization: macroscopic (the system in whole), mesoscopic (fiber contact area), and microscopic (structure of individual fibers) levels [56], and structural organization at all levels is controlled by the regime of electrospinning and composition of feed solution. For the *Hmi*/PHB composite, the introduction of hemin molecules to the feed mixture appears to exert a marked effect on the structural organization of *Hmi*/PHB at all levels.

At the stage of the electrospinning, the introduction of an even concentration of *Hmi* (1, 3, 5 wt.%) to the *Hmi*/PHB cocktail provides a sustainable regime of ES and leads to the formation of regular nonwoven fibrous material with a uniform distribution of *Hmi* within the amorphous phase between crystallites [51] as proved by the EDX method. The role of *Hmi* is likely to be associated with an increased electrical conductivity of the feed solution due to the presence of the metal ion as the coordination center in the *Hmi* molecule and the viscosity of the solution [57]. As a result, the electrospinning conditions are improved, and the performance of ES is enhanced. As the content of *Hmi* in the *Hmi*/PHB solution increases (from 1 to 5 wt.%), the fibrous material becomes more uniform, and the thickness of regular fibers without defects (smudges, spindles, etc.) decreases down to 1.77 µm. Noteworthy is that the fibers are well developed upon the Taylor cone formation stage [50]. In this case, due to the well-organized movement of a jet upon ES, the porosity of the *Hmi*/PHB material increases from 80 (pristine PHB) to 94% (*Hmi*/PHB containing 5 wt.%). In other words, at the high content of *Hmi* (Table 1), the *Hmi*/PHB material is characterized by a high percentage of the air layer between well-cured fibers.

Concerning the effect of hemin on the morphology of the *Hmi*/PHB materials, the SEM observations (Figure 3) prove that the quality and uniform character of the resultant *Hmi*/PHB nonwoven materials are improved due to the presence of big-sized *Hmi* molecules in the intermolecular space of PHB; as a result, the content of amorphous regions increases, thus increasing the rate of solvent desorption. Evidently, the above changes are provided by the supramolecular structure of PHB [44]. Electrospinning is known to be accompanied by a high deformation rate of the feed polymer solution (103 s^−1^) [51], thus governing the orientation of polymer chains [58]. Upon electrospinning, PHB macromolecules are organized as fibers containing alternating crystalline and amorphous regions [59]. Without the action of external forces, spherulites are formed.

As follows from the DSC scans (Figure 6b), with increasing the content of *Hmi* in the *Hmi*/PHB materials (from 0 to 5 wt.%), the melting peak becomes sharper and the onset of melting is shifted to higher temperatures. This evidence proves the formation of more regular crystallites with regular average dimensions and the absence of poorly organized small crystallites. However, the overall degree of crystallinity decreases (from 65% for neat PHB down to 53% for *Hmi*/PHB containing 5 wt.% of *Hmi*). Hemin is likely to serve as nucleation sites for the formation of uniform PHB crystallites; after crystallization, *Hmi* is accommodated within the amorphous phase of the *Hmi*/PHB materials. According to the EPR observations, as the content of *Hmi* in the *Hmi*/PHB samples increases, correlation time markedly decreases (Figure 8).

The above changes in the morphology of the *Hmi*/PHB materials are associated with the mechanical properties of the resultant materials. The addition of *Hmi* to the feed mixtures provides the formation of regular and uniform fibers and better-organized and well-cured nonwoven materials. The fibers contain no defects as bonds, spindles, smudges, and thinned regions, and their thickness is uniform along their length. Hence, the mechanical characteristics of the *Hmi*/PHB materials are improved.

The stability of the resultant *Hmi*/PHB materials was tested under the conditions of natural weathering (outdoor exposure). In general, weathering of PHB is accompanied by diverse processes, including oxidation, hydrolysis, photooxidation, stress-induced changes, etc., which lead to degradation and cleavage of the polymer chain to shorter fragments [60]. Degradation of biodegradable polymers strongly depends on many factors, including microbial activity, polymer composition, molecular weight, degree of crystallinity, temperature, humidity, pH, UV action, accessibility to oxygen and other oxidizing agents, etc. [61]. According to general knowledge, biodegradation is primarily provided by the biological activity of living organisms and leads to the decomposition of organic compounds into nontoxic products with lower molecular weights. Biodegradation of PHB under anaerobic conditions is accompanied by the formation of carbon dioxide (CO_2_), water (H_2_O), and methane; under aerobic conditions, CO_2_ and H_2_O are formed [62,63,64]. Several bacteria and fungi, e.g., *Pseudomonas*, *Actinomadura*, *Penicillium Aspergillus* spp., *Microbispora*, *Saccharomonospora*, *Streptomyces*, *Thermoactinomyces,* and *Bacillusspp.*, can also degrade PHAs both aerobically and anaerobically. Degradation of ultrathin PHB fibers is known to proceed with a high rate as compared with that of PHB films [65] due to the three-dimensional structure and high surface area of nanofibers [65,66].

The results on the outdoor weathering of the *Hmi*/PHB nonwoven materials containing thin fibers seem to be of vital importance for the characterization of their stability and potential use in biomedical applications. As follows from Figure 9, even after outdoor exposure for 90 days, the weathered *Hmi*/PHB samples preserve their shape and integrity. Further tests reveal insignificant fiber cleavage and thinning. The FTIR analysis reveals that degradation proceeds without any accumulation of functional groups or toxic products. As follows from Table 4, this process leads to certain deterioration in the mechanical properties of the *Hmi*/PHB materials: tensile strength decreases by a factor of 2, and elongation at break decreases by a factor of 1.5 (Figure 11). This evidence can be explained by the degradation-induced changes in the integrity of fibers and supramolecular structure of the *Hmi*/PHB materials due to oxidative and photooxidative processes in the air under the action of the UV light. Earlier, this phenomenon was observed for ozone-induced oxidation of PHB [67].

The DSC data [68] were analyzed for the detailed description of the structural evolution in the crystalline phase as evidenced by marked changes in physicomechanical properties.

Within 90 days of exposure, the supramolecular structure (including the crystalline phase) experiences marked changes in the action of environmental factors (water, wind, UV, temperature changes). The slight decrease in the melting temperature of the weathered samples suggests the formation of poorly organized crystallites and a certain decrease in the molecular weight of the samples [69,70]. However, in general, degradation of the *Hmi*/PHB materials is retarded as compared with the neat PHB. Seemingly, the exposure to solar radiation leads to the initiation of oxidative processes on the surface of individual fibers, leading to minor changes in the crystalline structure as evidenced by a well-pronounced shoulder and double melting peaks which are indicative of the formation of imperfect crystallites. Noteworthy is that the overall degree of crystallinity remains unchanged.

Let us also mention the action of *Hmi* as an efficient antibacterial agent with respect to bacteria and fungi [34,71,72,73]. In the presence of *Hmi*, intensive generation of hydroxyl radical leads to oxidation of cellular compartments, cell wall rupturing, and membrane leakage, thus providing bacterial apoptosis. As a result, the amorphous phase of the *Hmi*/PHB composite materials, which is the most susceptible to biodegradation, appears to be stabilized. Hence, the resultant *Hmi*/PHB composites with high biocompatibility, good mechanical properties, and retarded biodegradation can be used as effective biomedical materials and scaffolds for regenerative medicine and tissue engineering.

## 5. Conclusions

This work addresses the preparation of bioinspired *Hmi*/PHB composite materials with high mechanical properties and retarded biodegradation. The addition of *Hmi* to the feed PHB solution (above 3 wt.%) provides a marked improvement in the performance of the electrospinning process and leads to the formation of well-organized and uniform nonwoven materials with high porosity and good mechanical properties.

The work investigated and described the regularities of changes in the PHB-hemin system during outdoor exposure. The obtained results revealed a deeper understanding of the structural organization of PHB macromolecules and their response to oxidative processes. In the case of the commercial launch of the material, practical recommendations for the use of the PHB-hemin system can be carried out. This material retains its stability within 90 days. The *Hmi*/PHB materials can be recommended for their use as biomedical materials for wound disinfection and accelerated healing, and as scaffolds in regenerative medicine and tissue engineering, and this challenging task requires further detailed studies.

## Figures and Tables

**Table 1:** Structural characteristics of PHB and *Hmi*/PHB nonwoven composites.

Content of *Hmi* %	Density (Mean ± SD, *n* = 10) g/cm^3^	Average Diameter of Fibers (Mean ± SD, *n* = 100) µm	Porosity (Mean ± SD, *n* = 10) %
0	0.30 ± 0.01	3.50 ± 0.08	80 ± 2.0
1	0.20 ± 0.02	2.06 ± 0.07	92 ± 1.5
3	0.20 ± 0.01	1.77 ± 0.04	92 ± 1.5
5	0.17 ± 0.01	1.77 ± 0.04	94 ± 1.2

**Table 2:** Mechanical characteristics of the *Hmi*/PHB samples with different content of *Hmi*.

Content of *Hmi* %	Tensile Strength (Mean ± SD = 0.05, n = 10) MPa	Elongation at Break (Mean ± SD = 0.2, n = 10) %
0	1.7	3.6
1	0.7	4.7
3	1.9	4.7
5	5.5	6.1

**Table 3:** Thermophysical characteristics (T_m_—the melting temperature, Δ*H*—the heat of fusion) of weathered PHB and *Hmi*/PHB nanocomposites (90 days of exposure).

Sample	Exposure Time Days	First Heating Run		Second Heating Run	
		T_m_, °C	Δ*H*, J/g	T_m_, °C	Δ*H*, J/g
*Hmi*/PHB (5 wt.%)	0	174.5	88.7	171.0	87.9
*Hmi*/PHB (5 wt.%)	90	171.3	82.8	166.9	80.2
PHB	0	174.1	73.6	170.4	79.4
PHB	90	170.8	63.2	167.2	64.9

**Table 4:** Mechanical characteristics of the neat and weathered *Hmi*/PHB materials (90 days of exposure).

Content of *Hmi* %	Exposure Time Days	Tensile Strength (Mean ± SD = 0.05, *n* = 10) MPa	Elongation at Break (Mean ± SD = 0.2, *n* = 10) %
5	0	5.5	6.1
5	90	1.9	4.5
0	0	1.7	3.6
0	90	2.7	4.2

## Supplementary Materials

The following supporting information can be downloaded at: https://www.mdpi.com/article/10.3390/polym14224878/s1, Figure S1: Photo and schematic view of the single-capillary laboratory unit for the electrospinning process; Figure S2: Histogram the fibers’ size distribution of PHB and *Hmi*/PHB nonwoven composites.
