# Supplementary material for: Bioinspired Electropun Fibrous Materials Based on Poly-3-Hydroxybutyrate and Hemin: Preparation, Physicochemical Properties, and Weathering
Source: Polymers (Basel). 2022 Nov 12;14(22):4878. doi: 10.3390/polym14224878 (PMC9692885; doi:10.3390/polym14224878)
Supplement: Supplementary file 1 [file polymers-14-04878-s001.zip › polymers-1960294-supplementary.pdf]

Figure S1. Photo (a) and schematic view of the single-capillary laboratory unit for the electrospinning process (b), where: 1 – protective installation box; 2 – bin with a polymer solution and a capillary; 3 – high voltage source; 4 – stable precipitating electrode, 5 – air pressure regulator.

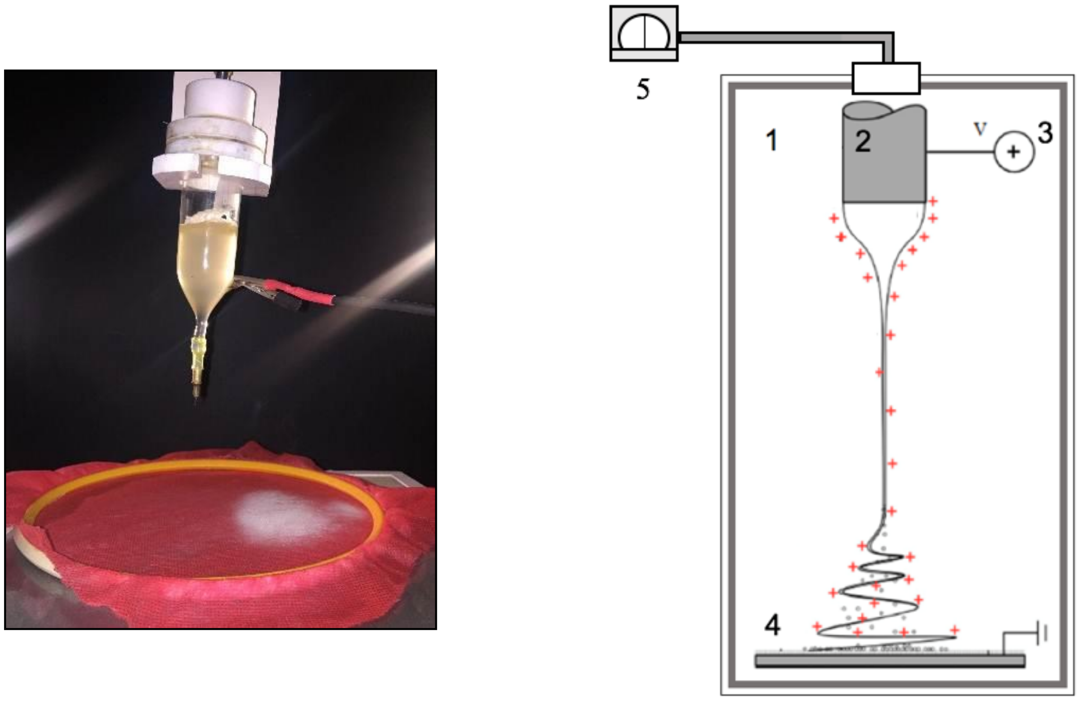

(a) **(b)**

Figure S2. Histogram the fibers' size distributionof PHB and H_{mi}/PHB nonwoven composites, where: a – 0 wt. %, b – 1 wt. %, c – 3 wt.%, and d – 5wt.%.

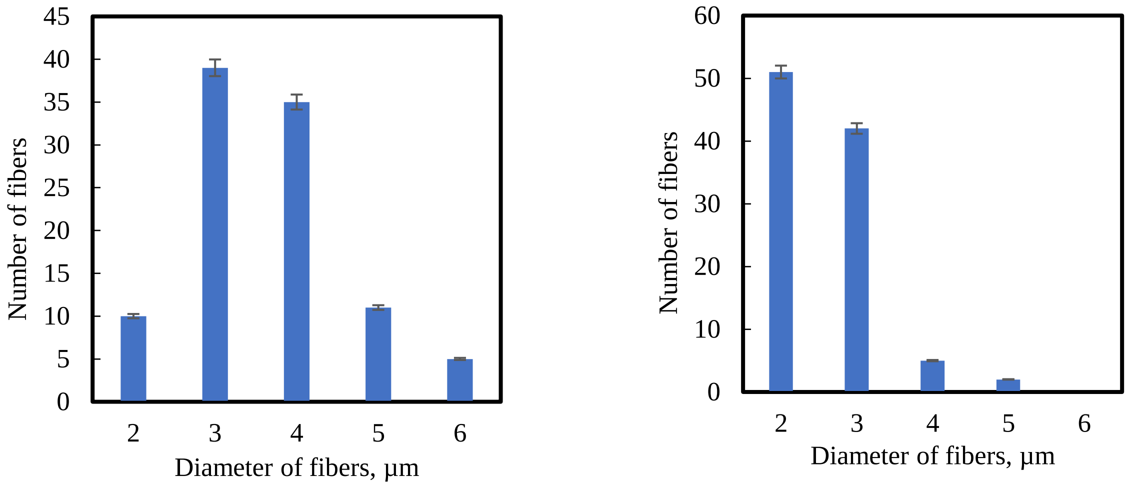

(a) **(b)**

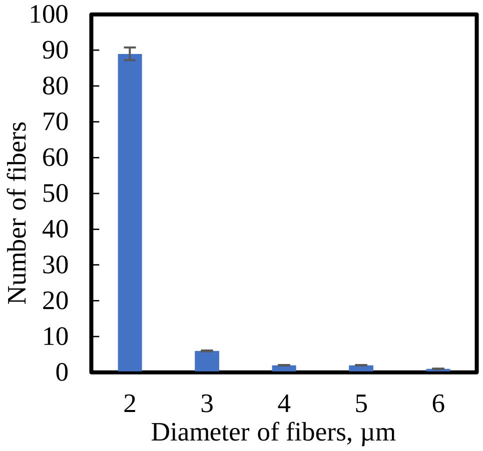
100
90
80
70
60
50
40
30
20
10
0
Number of fibers
2
3
4
5
6
Diameter of fibers, µm

(c)

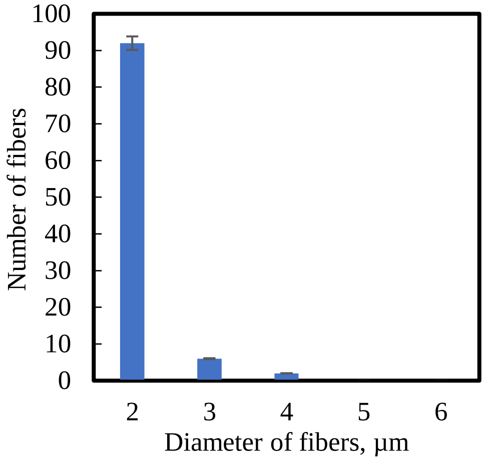
100
90
80
70
60
50
40
30
20
10
0
Number of fibers
2
3
4
5
6
Diameter of fibers, µm

(d)
